# Fluctuating selection on basal metabolic rate

**DOI:** 10.1002/ece3.1954

**Published:** 2016-01-25

**Authors:** Johan F. Nilsson, Jan‐Åke Nilsson

**Affiliations:** ^1^ Department of Biology, Evolutionary EcologyLund UniversityEcology BuildingSE‐223 62LundSweden

**Keywords:** Basal metabolic rate, fluctuating selection, metabolic strategy, winter survival

## Abstract

BMR (Basal metabolic rate) is an important trait in animal life history as it represents a significant part of animal energy budgets. BMR has also been shown to be positively related to sustainable work rate and maximal thermoregulatory capacity. To this date, most of the studies have focused on the causes of interspecific and intraspecific variation in BMR, and fairly little is known about the fitness consequences of different metabolic strategies. In this study, we show that winter BMR affects local survival in a population of wild blue tits (*Cyanistes caeruleus*), but that the selection direction differs between years. We argue that this fluctuating selection is probably a consequence of varying winter climate with a positive relation between survival and BMR during cold and harsh conditions, but a negative relation during mild winters. This fluctuating selection can not only explain the pronounced variation in BMR in wild populations, but will also give us new insights into how energy turnover rates can shape the life‐history strategies of animals. Furthermore, the study shows that the process of global warming may cause directional selection for a general reduction in BMR, affecting the general life‐history strategy on the population level.

## Introduction

BMR (Basal metabolic rate) is probably one of the most commonly measured physiological traits in endothermic animals. It represents the minimal energy expenditure for a resting, fasting animal at thermoneutrality and thus represents the lowest sustainable energetic cost of running and maintaining the body (McNab 2002). In most cases, BMR represents a significant proportion of animal energy budgets (Speakman 2000; Burton et al. 2011), as well as being positively related to sustained peak work rate (Hammond and Diamond 1997; Nilsson 2002), and is therefore an important trait in the life of endothermic animals.

In most species, intraspecific variation in BMR is fairly large, but individual BMR is generally repeatable over both short and long timescales (Labocha et al. 2004; Rønning et al. 2005; Broggi et al. 2009), indicating a consistent interindividual variation. This consistency in individual levels of BMR may depend on both genetic and nongenetic effects. Both of these have been demonstrated to influence BMR, for example, rearing conditions (Verhulst et al. 2006), maternal effects (Tobler et al. 2007; Nilsson et al. 2011), and heritability (Konarzewski et al. 2005; Rønning et al. 2007; Nilsson et al. 2009; Wone et al. 2009; Careau et al. 2011).

Common garden experiments have shown that the differences in BMR between populations have a strong genetic component (Wikelski et al. 2003; Broggi et al. 2005). Moreover, a selection experiment in mice has shown that BMR responds to artificial selection (Ksiazek et al. 2004; Gebczynski and Konarzewski 2009). This shows that BMR has a genetic component and thus that the average trait value could also change in the wild due to the selection. However, as is the case for many physiological traits, BMR also exhibits a great deal of phenotypic plasticity (Tieleman et al. 2003; Swanson 2010). This phenotypic plasticity is manifested both in reversible intraindividual seasonal variation in sedentary animals – for example, an increase in BMR during winter (McKechnie 2008) – and as short‐term adjustments in relation to immediate variation in the environment – for example, an increase in BMR in response to decreasing temperatures (Broggi et al. 2007; McKechnie 2008). Thus, each genotype can produce a range of phenotypic trait values in response to, for example, variable ambient temperature, that is, reaction norms. However, the variation in the shape of the reaction norm seems to also include additive genetic variation (Broggi et al. 2005; McKechnie 2008).

In both small birds and small mammals, BMR increases gradually with latitude (Wiersma et al. 2007; Raichlen et al. 2010; Smit and McKechnie 2010). Species living at high latitudes, in cold environments, generally have higher BMR than species living at low latitudes in hot and arid environments. This relation is also common within species (Broggi et al. 2004), and BMR is generally higher in winter than in summer in at least some small temperate birds (Cooper 2002; Liknes et al. 2002; McKechnie 2008; Swanson 2010). This increase in BMR in cold environments has been suggested to be functionally correlated with improved cold resistance and to be part of the winter acclimatisation process (Liknes and Swanson 1996; White et al. 2007; Smit and McKechnie 2010). Moreover, a direct, positive relation between seasonally increased BMR and temperature‐induced summit metabolism is usually also found (Liknes and Swanson 1996; Liknes et al. 2002). Furthermore, experimentally induced reductions in ambient temperature generally result in an increase in BMR after some acclimation time (McKechnie 2008; Caro and Visser 2009), a relation that also holds within individuals (McKechnie et al. 2007). Thus, it seems that decreasing ambient temperatures, calling for increased capacity for thermogenesis, commonly result in an increased BMR. Individuals with a high BMR also have a better capacity to respond to a sudden decrease in temperature and still maintain energy balance than individuals with a low BMR (Ksiazek et al. 2009).

Given the pronounced portion of the total energy turnover rate that can be ascribed to BMR (e.g., thermoregulation during the night at a temperature of 0°C in blue tits and −10°C in great tits (*Parus major*) increases the metabolic rate by 0.6 and 0.8 times BMR, respectively) (Nilsson and Svensson 1996; Broggi et al. 2004), it is reasonable to expect that variation in BMR will lead to fitness differences. Fitness consequences of interindividual variation in BMR have received comparatively little attention, especially in natural conditions (Burton et al. 2011). In the few studies looking for a relation between survival and BMR, both negative (Larivée et al. 2010) and positive (Jackson et al. 2001) relations have been found. Together with the large variation between individuals found in many populations (Versteegh et al. 2008), this could indicate either weak selection on BMR or spatially or temporally varying selection pressures. The link between BMR and fitness has also been proposed to be context dependent, possibly allowing different metabolic phenotypes to be favoured depending on the prevailing conditions (Burton et al. 2011).

In this study, we investigated how winter BMR affected the survival from the winter to the following breeding season in a wild population of blue tits. Many animals experience highest mortality during the winter months, and as environmentally induced metabolic requirements increase during the winter, we predict that variation in BMR should have a pronounced effect on survival.

## Materials and Methods

The study was conducted on a population of free‐living blue tits in southern Sweden (55° 42′ N, 13° 28′ E) between 2004 and 2006. Temperature data were obtained from a climate station in the middle of the study area. Birds were caught during winter while roosting in nest boxes, and BMR was measured the same night as the overnight oxygen consumption (VO_2_) in an open‐circuit respirometer. The metabolic measurements lasted until the morning (before dawn) to ensure that the BMR measurement was taken when the birds were in a postabsorptive state. Each bird was placed in a sealed Plexiglas^®^ chamber (1.6 L) and then placed in the darkness of a climate cabinet (type VEM 03/500, Heraeus Vötsch, Hanau, Germany) at 25°C, that is, within their thermoneutral zone. The respirometer consisted of two identical blocks, each with a carbon dioxide analyser and an oxygen analyser. Within a block, an automatic valve control system was used to switch between the two chambers and baseline every 20 min, thus allowing the measurement of four birds per night (two per block). All chambers had their own pump, ensuring an even airflow through the chambers at all times. Air was pulled from the respirometer chamber and dried with silica gel before passing through the carbon dioxide analyser (Servomex 1440 two‐channel analyzer, Crowborough, U.K.), after which it passed through the oxygen analyser (Servomex 4100 two‐channel analyser, Crowborough, U.K.). The carbon dioxide analyser was zero‐calibrated with pure nitrogen (99.998% N_2_). The oxygen analyser was also zero‐calibrated with pure nitrogen and then calibrated with dry ambient air to 20.95% oxygen before measurements started each evening. High‐precision flow meters (Bronkhorst HI‐TEC, Ruurlo, the Netherlands), positioned before the carbon dioxide analyser, were used to adjust the flow rate to 166 mL min^−1^. This flow rate was chosen because, assuming an oxygen consumption of 0.77 mL O_2_ min^−1^ (Nilsson and Svensson 1996), it would reduce the proportion of oxygen in excurrent air to 20.5% and would thereby be within the range of best accuracy of the oxygen analyser. Oxygen and carbon dioxide concentrations were automatically recorded on a Squirrel data logger (model 1202, Grant instruments, Cambridge, U.K.) every minute throughout the measurement sessions. VO_2_ and VCO_2_ were calculated according to the equation in Withers (2001), accounting for the fact that CO_2_ was not scrubbed prior to the measurements. The lowest 10‐min running average of VO_2_ was used as a measure of BMR, excluding the first two minutes in all measurement cycles to allow the flushing of the respirometer. The RQ (respiratory quotient) was calculated as RQ = VCO_2_/VO_2_. Before dawn, all birds were returned to the nest box where they had been caught, to minimise any potential disturbance. All birds were ringed with individually numbered aluminum rings. No birds in the study were sampled more than once.

Birds that were recaptured in the population during the following breeding season or later (until winter 2008) were categorised as survivors. Birds that were not resighted could have either died or dispersed. However, dispersal is unlikely as the study was conducted during winter and most blue tit dispersal occurs during the autumn. Furthermore, from the distribution of ages and sexes among the birds roosting in the nest boxes compared with the population in general, we concluded that it is the most dominant segment of the population that has access to the boxes. This is further strengthened by actual spring dispersal distances from roosting to breeding nest box as 63% of first‐year breeders were found to breed in the same nest box pair and 86% bred within 300 m of their roosting box. The longest dispersal distance between roosting box and breeding box was 2300 m. In a study site of 64 km^2^, this implies that few roosting birds subsequently dispersed to other areas before breeding. The density of the boxes in our study area was low; thus, some blue tits bred in natural cavities within the study area. We assumed that the sample of blue tit pairs breeding in the boxes is random in relation to BMR. The fixed number of nest boxes in combination with a preference for nest boxes over natural cavities also results in low variation in the number of nest box occupants between years. During the winter 2004–2005, birds were caught between 27th of December and 3rd of February; during the winter 2005–2006, birds were caught between 1st of December and 21st of February.

All statistics were calculated in SAS 9.3(SAS Institute Inc., Cary, NC, USA). A general linear mixed model was used to calculate BMR residuals (BMR as a dependent factor, body mass as a covariate, and respirometer channel as a random factor). The Satterthwaite approximation was used to calculate the denominator degrees of freedom. A linear probability model (SAS GENMOD with logit function) was used to model the survival probability in relation to BMR residuals, age, sex, tarsus length, year, and date of capture. All two‐way interactions were included in the initial model. Factors, covariates, and interactions with *P* > 0.1 were removed from the final model.

## Results

**Figure 1:**
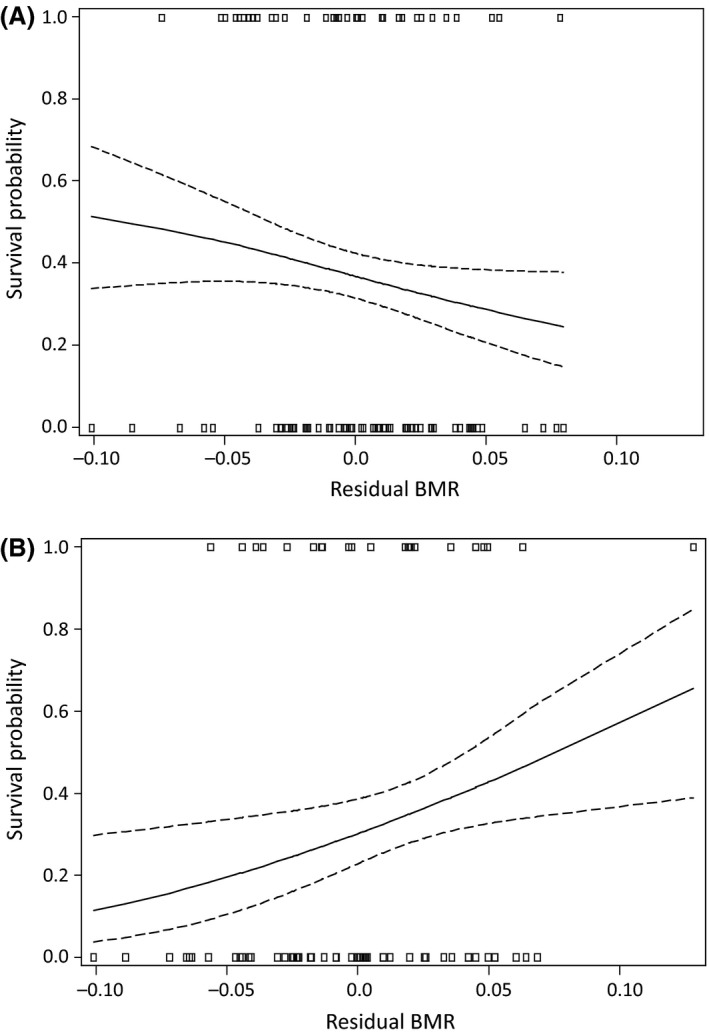
Survival probability in relation to basal metabolic rate between years. Survival of blue tits from the winters 2004/2005 (A) and 2005/2006 (B) until the next breeding season, as a function of BMR (basal metabolic rate). Squares represent actual survival (1 = survived). The lines represent survival probabilities for residual BMR (cubic splines, thick solid line ± SE, broken line) controlling for the effects of respirometer channel (random factor) and mass (covariate).

In total, 167 individuals were included in the study (2004–2005: 92 birds; 2005–2006: 75 birds). Residual BMR was used to control for the factors affecting winter BMR in the survival analysis (respirometer channel as random factor and mass as a covariate; mass: *F*_1,162_ = 152.94, *P* < 0.001). Survival was related to residual BMR, but the direction of selection differed between the two winters (GENMOD: residual BMR × year: *χ* ^2^ = 4.59; *P* = 0.032). In the winter 2004–2005 (Fig. 1A), selection favoured the birds with low residual BMR (regression coefficient: 7.44 ± 6.74), while in 2005–2006 (Fig. 1B), it favoured the birds with relatively high residual BMR (regression coefficient: 11.69 ± 6.44). The overall effect of residual BMR for the two winters combined was nonsignificant (residual BMR: *χ* ^2^ = 0.31; *P* = 0.58). Survival probability did not differ between the two winters (*χ* ^2^ = 1.85; *P* = 0.17), but males and older birds were more likely to survive than females and first‐year birds (sex: *χ* ^2^ = 4.58; *P* = 0.032, age: *χ* ^2^ = 5.53; *P* = 0.019).

Temperature characteristics differed between the two winters (Table 1). The first winter was quite mild and had only a few days with snow cover, while the second winter was colder and with snow cover lasting for several weeks. Whereas snow coverage does not affect the thermoregulatory costs, it can potentially affect foraging costs negatively, thereby adding to the costs of the lower temperature.

**Table 1:** Climate characteristics of the two winters included in the study. Mean (±SE) daily average and minimum temperature (°C) as well as the number of days with a mean average daily temperature below 0.0°C for the period 1 December–28 February in the winters 2004/2005 and 2005/2006. The normal mean temperature (WMO: 1961–90) for the period was 0.0°C

	Mean (±SE) temp. (°C)	Mean (±SE) min. temp. (°C)	Days with mean temp. below 0.0°C
Winter 2004–2005	1.96 ± 0.53	−0.41 ± 0.62	10
Winter 2005–2006	− 0.46 ± 0.30	−3.06 ± 0.42	20

## Discussion

Here, we show that BMR is related to winter survival in a wild population of blue tits and that the direction of this direct or indirect selection can vary between years. Although we can only speculate about the underlying causes of this pattern, it seems likely that this fluctuating selection could be explained by differences in meteorological conditions in the two winters (Table 1). Metabolic requirements are known to increase during the winter (Swanson 2010) at the same time as food availability and time for acquiring the food decrease. This induces a strong selection pressure for utilising energy in an efficient way, and even small differences in the pattern of energy acquisition and allocation are likely to have substantial fitness effects. In the temperate region, thermoregulation makes up a large part of the daily energy budget during winters and the cost increases rapidly with decreasing ambient temperatures (McNab 2002; McKechnie 2008). In contrast to mammals, birds only thermoregulate by shivering their muscles (especially the large pectoral muscle), and in line with this, several studies have found a link between BMR and maximal thermoregulatory capacity (Liknes and Swanson 1996; Liknes et al. 2002). A similar link has been found between BMR and susMR (sustainable metabolic rate) (Nilsson 2002; Tieleman et al. 2008; Sears et al. 2009). An increase in maximal thermoregulatory capacity or susMR requires an increased mass of energy‐supplying organs such as intestines, liver, kidney, and heart as well as increased muscle mass to sustain shivering thermogenesis (Raichlen et al. 2010; Zhang et al. 2015). These organs and muscles are also very metabolically active, establishing the functional link between BMR and susMR (Nilsson 2002; Raichlen et al. 2010). Selection on these energy‐supplying organs is probably the reason for observed variation in BMR in cold environments.

During the second winter of this study, the mean temperature was lower (Table 1) and consequently the need for high cold resistance and the resulting thermoregulatory costs were higher than during the previous winter. During these conditions, it is therefore not surprising that birds with high BMR survive better (Fig. 1B), because the high BMR allows them to have a high capacity of thermogenesis. As has been argued for selection on personalities (Careau et al. 2008), in situations when a high susMR is needed, selection will indirectly favour individuals with a high BMR. This selection pressure will, however, potentially be lessened when the need of a high susMR or high thermogenesis capacity is relaxed. High‐BMR individuals could then instead be selected against, as they still have to pay the cost of higher energetic needs without the advantage of a need for increased thermoregulatory capacity. Thus, during a mild winter, as in the first year of the study, there is no need for high rates of thermogenesis and a high BMR would then be disadvantageous as it only leads to costs in the form of increased food requirements and potentially increased risks of predation. This is in line with the generally observed reduction in BMR at southern latitudes, with a reduced need for high rates of thermogenesis, compared to more northerly latitudes although ambient temperatures may be well below the thermoneutral zone in both areas (Broggi et al. 2004).

We suggest that the temporally varying selection pattern that we found, together with a trait heritability of 0.59 in this population (Nilsson et al. 2009), will produce a substantial variation in BMR in the wild. In line with our study is the finding that the relation between reproductive success and metabolic rate of bank voles (*Myodes glareolus*) also varies between seasons (Boratyn'ski and Koteja 2010). These examples of fluctuating selection, together with the evidence that selection on BMR can be sex dependent (Boratyn'ski and Koteja 2010; Boratyn'ski et al. 2010), corroborate the recently proposed “context dependence” hypotheses for the relation between BMR and fitness (Burton et al. 2011). Fluctuating selection could not only explain the pronounced variation in BMR in wild populations, but will also give us new insights into how energy turnover rates can shape the life‐history strategies of animals. Because the selection direction seems to depend on the winter conditions in our case, the favoured metabolic phenotype will vary between years. As BMR has been suggested to be intimately related to life‐history variation, for example, in reproductive strategies along a fast–slow axis (Wikelski et al. 2003), this variation may be predicted to also differ between years according to the selective process during the previous winter. Furthermore, in a global warming scenario, selection on winter survival may result in a directional reduction in BMR, potentially indirectly affecting the general life history in the population favouring low BMR genotypes with a slow rate of living.

## Conflict of Interest

None declared.

## References

[ece31954-bib-0001] Boratyn'ski, Z. , and P. Koteja . 2010 Sexual and natural selection on body mass and metabolic rates in free‐living bank voles. Funct. Ecol. 24:1252–1261.

[ece31954-bib-0002] Boratyn'ski, Z. , E. Koskela , T. Mappes , and A. T. Oksanen . 2010 Sex‐specific selection on energy metabolism ‐ selection coefficients for winter survival. J. Evol. Biol. 23:1969–1978. 20695968 10.1111/j.1420-9101.2010.02059.x

[ece31954-bib-0003] Broggi, J. , M. Orell , E. Hohtola , and J.‐Å. Nilsson . 2004 Metabolic response to temperature variation in the great tit: an interpopulation comparison. J. Anim. Ecol. 73:967–972.

[ece31954-bib-0004] Broggi, J. , E. Hohtola , M. Orell , and J.‐Å. Nilsson . 2005 Local adaptation to winter conditions in a passerine spreading north: a common‐garden approach. Evolution 59:1600–1603. 16153046

[ece31954-bib-0005] Broggi, J. , E. Hohtola , K. Koivula , M. Orell , R. L. Thomson , and J.‐Å. Nilsson . 2007 Sources of variation in winter basal metabolic rate in the great tit. Funct. Ecol. 21:528–533.

[ece31954-bib-0006] Broggi, J. , E. Hohtola , K. Koivula , M. Orell , and J.‐Å. Nilsson . 2009 Long‐term repeatability of winter basal metabolic rate and mass in a wild passerine. Funct. Ecol. 23:768–773. doi:10.1111/j.1365‐2435.2009.01561.x.

[ece31954-bib-0007] Burton, T. , S. S. Killen , D. J. Armstrong , and N. B. Metcalfe . 2011 What causes intraspecific variation in resting metabolic rate and what are its ecological consequences? Proc. Biol. Sci. 278:3465–3473. doi:10.1098/rspb.2011.1778. 21957133 10.1098/rspb.2011.1778 PMC3189380

[ece31954-bib-0008] Careau, V. , D. Thomas , M. M. Humphries , and D. Réale . 2008 Energy metabolism and animal personality. Oikos 117:641–653.

[ece31954-bib-0009] Careau, V. , D. Thomas , F. Pelletier , L. Turki , F. Landry , D. Garant , and D. Réale . 2011 Genetic correlation between resting metabolic rate and exploratory behaviour in deer mice (*Peromyscus maniculatus*). J. Exp. Biol. 24:2153–2163. 10.1111/j.1420-9101.2011.02344.x 21696480

[ece31954-bib-0010] Caro, S. P. , and M. E. Visser . 2009 Temperature‐induced elevation of basal metabolic rate does not affect testis growth in great tits. J. Exp. Biol. 212:1995–9. doi:10.1242/jeb.026344. 19525424 10.1242/jeb.026344

[ece31954-bib-0011] Cooper, S. J. 2002 Seasonal metabolic acclimatization in mountain chickadees and juniper titmice. Physiol. Biochem. Zool. 75:386–95. doi:10.1086/342256. 12324895 10.1086/342256

[ece31954-bib-0012] Gebczynski, A. K. , and M. Konarzewski . 2009 Locomotor activity of mice divergently selected for basal metabolic rate: a test of hypotheses on the evolution of endothermy. J. Evol. Biol. 22:1212–1220. 19344384 10.1111/j.1420-9101.2009.01734.x

[ece31954-bib-0013] Hammond, A. K. , and J. Diamond . 1997 Maximal sustained energy budgets in humans and animals. Nature 386:457–462. 9087402 10.1038/386457a0

[ece31954-bib-0014] Jackson, M. D. , P. Trayhurn , and R. J. Speakman . 2001 Associations between energetics and over‐winter survival in the short‐tailed field vole *Microtus agrestis* . J. Anim. Ecol. 70:633–640.

[ece31954-bib-0015] Konarzewski, M. , A. Ksiazek , and B. I. Lapo . 2005 Artificial selection on metabolic rates and related traits in rodents. Integr. Comp. Biol. 45:416–425. 21676787 10.1093/icb/45.3.416

[ece31954-bib-0016] Ksiazek, A. , M. Konarzewski , and B. I. Lapo . 2004 Anatomic and energetic correlates of divergent selection for basal metabolic rate in laboratory mice. Physiol. Biochem. Zool. 77:890–899. 15674764 10.1086/425190

[ece31954-bib-0017] Ksiazek, A. , J. Czerniecki , and M. Konarzewski . 2009 Phenotypic flexibility of traits related to energy acquisition in mice divergently selected for basal metabolic rate (BMR). J. Exp. Biol. 212:808–814. doi:10.1242/jeb.025528. 19251997 10.1242/jeb.025528

[ece31954-bib-0018] Labocha, K. M. , T. E. Sadowska , K. Baliga , A. K. Semer , and P. Koteja . 2004 Individual variation and repeatability of basal metabolism in the bank vole, *Clethrionomys glareolus* . Proc. Biol. Sci. 271:367–372. 15101695 10.1098/rspb.2003.2612 PMC1691610

[ece31954-bib-0019] Larivée, L. M. , S. Boutin , R. J. Speakman , A. G. McAdam , and M. M. Humphries . 2010 Associations between over‐winter survival and resting metabolic rate in juvenile North American red squirrels. Funct. Ecol. 24:597–607.

[ece31954-bib-0020] Liknes, T. E. , and L. D. Swanson . 1996 Seasonal variation in cold tolerance, basal metabolic rate, and maximal capacity for thermogenesis in white‐breasted nuthatches *Sitta carolinensis* and downy woodpeckers *Picoides pubescens*, two unrelated arboreal temperate residents. J. Avian Biol. 27:279–288.

[ece31954-bib-0021] Liknes, E. T. , S. M. Scott , and D. L. Swanson . 2002 Seasonal acclimatization in the American goldfinch revisited: to what extent do metabolic rates vary seasonally? Condor 104:548. doi:10.1650/0010‐5422(2002)104[0548:SAITAG]2.0.CO;2.

[ece31954-bib-0022] McKechnie, E. A. 2008 Phenotypic flexibility in basal metabolic rate and the changing view of avian physiological diversity: a review. J. Comp. Physiol. B. 178:235–247. 17957373 10.1007/s00360-007-0218-8

[ece31954-bib-0023] McKechnie, E. A. , K. Chetty , and G. B. Lovegrove . 2007 Phenotypic flexibility in the basal metabolic rate of laughing doves: responses to short‐term thermal acclimation. J. Exp. Biol. 210:97–106. 17170153 10.1242/jeb.02615

[ece31954-bib-0024] McNab, K. B. 2002 The physiological ecology of vertebrates: a view from energetics. Cornell Univ. Press, New York.

[ece31954-bib-0025] Nilsson, J.‐Å. 2002 Metabolic consequences of hard work. Proc. Biol. Sci. 269:1735–1739. 12204136 10.1098/rspb.2002.2071 PMC1691085

[ece31954-bib-0026] Nilsson, J.‐Å. , and E. Svensson . 1996 The cost of reproduction: a new link between current reproductive effort and future reproductive success. Proc. Biol. Sci. 263:711–714.

[ece31954-bib-0027] Nilsson, J.‐Å. , M. Åkesson , and F. J. Nilsson . 2009 Heritability of resting metabolic rate in a wild population of blue tits. J. Evol. Biol. 22:1867–1874. 19682309 10.1111/j.1420-9101.2009.01798.x

[ece31954-bib-0028] Nilsson, F. J. , M. Tobler , J.‐Å. Nilsson , and I. M. Sandell . 2011 Long‐lasting consequences of elevated yolk testosterone for metabolism in the zebra finch. Physiol. Biochem. Zool. 84:287–291. 21527819 10.1086/659006

[ece31954-bib-0029] Raichlen, D. A. , A. D. Gordon , M. N. Muchlinski , and J. J. Snodgrass . 2010 Causes and significance of variation in mammalian basal metabolism. J. Comp. Physiol. B. 180:301–311. 19730868 10.1007/s00360-009-0399-4

[ece31954-bib-0030] Rønning, B. , B. Moe , and C. Bech . 2005 Long‐term repeatability makes basal metabolic rate a likely heritable trait in the zebra finch *Taeniopygia guttata* . J. Exp. Biol. 208:4663–4669. 16326947 10.1242/jeb.01941

[ece31954-bib-0031] Rønning, B. , H. Jensen , B. Moe , and C. Bech . 2007 Basal metabolic rate: heritability and genetic correlations with morphological traits in the zebra finch. J. Evol. Biol. 20:1815–1822. 17714299 10.1111/j.1420-9101.2007.01384.x

[ece31954-bib-0032] Sears, M. W. , P. J. Hayes , R. M. Banta , and D. McCormick . 2009 Out in the cold: physiological capacity influences behaviour in deer mice. Funct. Ecol. 23:774–783.

[ece31954-bib-0033] Smit, B. , and A. E. McKechnie . 2010 Avian seasonal metabolic variation in a subtropical desert: basal metabolic rates are lower in winter than in summer. Funct. Ecol. 24:330–339. doi:10.1111/j.1365‐2435.2009.01646.x.

[ece31954-bib-0034] Speakman, R. J. 2000 The cost of living: field metabolic rates of small mammals. Adv. Ecol. Res. 30:177–297.

[ece31954-bib-0035] Swanson, D. L. 2010 Seasonal metabolic variation in birds: functional and mechanistic correlates. Curr. Ornithol. 17:75–129.

[ece31954-bib-0036] Tieleman, I. B. , E. M. Buschur , and R. C. Brown . 2003 Phenotypic variation of larks along an aridity gradient: are desert birds more flexible? Ecology 84:1800–1815.

[ece31954-bib-0037] Tieleman, I. B. , H. T. Dijkstra , C. K. Klasing , G. H. Visser , and J. B. Williams 2008 Effects of experimentally increased costs of activity during reproduction on parental investment and self‐maintenance in tropical house wrens. Behav. Ecol. 19:949–959.

[ece31954-bib-0038] Tobler, M. , J.‐Å. Nilsson , and F. J. Nilsson . 2007 Costly steroids: egg testosterone modulates nestling metabolic rate in the zebra finch. Biol. Lett. 3:408–410. 17456447 10.1098/rsbl.2007.0127 PMC2390662

[ece31954-bib-0039] Verhulst, S. , M.‐J. Holveck , and K. Riebel . 2006 Long‐term effects of manipulated natal brood size on metabolic rate in zebra finches. Biol. Lett. 2:478–480. 17148435 10.1098/rsbl.2006.0496 PMC1686193

[ece31954-bib-0040] Versteegh, A. M. , B. Helm , J. N. Dingemanse , and I. B. Tieleman . 2008 Repeatability and individual correlates of basal metabolic rate and total evaporative water loss in birds: a case study in European stonechats. Comp. Biochem. Physiol. A 150:452–457. 10.1016/j.cbpa.2008.05.006 18571446

[ece31954-bib-0041] White, C. R. , T. M. Blackburn , G. R. Martin , and P. J. Butler . 2007 Basal metabolic rate of birds is associated with habitat temperature and precipitation, not primary productivity. Proc. R. Soc. B Biol. Sci. 274:287–293. 10.1098/rspb.2006.3727 PMC1685844 17148258

[ece31954-bib-0042] Wiersma, P. , A. Muñoz‐Garcia , A. Walker , and B. J. Williams . 2007 Tropical birds have a slow pace of life. Proc. Natl Acad. Sci. USA 104:9340–9345. 17517640 10.1073/pnas.0702212104 PMC1890496

[ece31954-bib-0043] Wikelski, M. , L. Spinney , W. Schelsky , A. Scheuerlein , and E. Gwinner . 2003 Slow pace of life in tropical sedentary birds: a common‐garden experiment on four stonechat populations from different latitudes. Proc. Biol. Sci. 270:2383–2388. 14667355 10.1098/rspb.2003.2500 PMC1691521

[ece31954-bib-0044] Withers, P. C. 2001 Design, calibration and calculation for flow‐through respirometry systems. Aust. J. Zool. 49:445–461.

[ece31954-bib-0045] Wone, B. , M. W. Sears , K. M. Labocha , E. R. Donovan , and J. P. Hayes . 2009 Genetic variances and covariances of aerobic metabolic rates in laboratory mice. Proc. Biol. Sci. 276:3695–3704. 19656796 10.1098/rspb.2009.0980 PMC2817310

[ece31954-bib-0046] Zhang, Y. , K. Eyster , J.‐S. Liu , and D. L. Swanson . 2015 Cross‐training in birds: cold and exercise training produce similar changes in maximal metabolic output, muscle masses and myostatin expression in house sparrows, *Passer domesticus* . J. Exp. Biol. 218:2190–2200. doi:10.1242/jeb.121822. 25987736 10.1242/jeb.121822 PMC4528703

